# Good Behavior Game: Effects on Disruptive Behaviors of Students with and Without Special Educational Needs in Inclusive Setting

**DOI:** 10.3390/bs15020177

**Published:** 2025-02-08

**Authors:** Emrah Gulboy, Hicran Denizli-Gulboy, Salih Rakap

**Affiliations:** 1 Department of Specialized Education, Developmental Education Research and Practice Center (OGEM), Ondokuz Mayıs University, 55100 Samsun, Türkiye; 2 Department of Specialized Education, Eskişehir Osmangazi University, 26040 Eskişehir, Türkiye; hdenizliglby@gmail.com; 3 Department of Specialized Education Services, University of North Carolina, Greensboro, NC 27402, USA

**Keywords:** Good Behavior Game, classroom management, disruptive behaviors, students with special education needs, inclusive education

## Abstract

The Good Behavior Game (GBG) is a practical and evidence-based classroom management strategy that requires minimal time and resources to learn and implement. This study investigates the effectiveness of the GBG in reducing disruptive behaviors among students with special educational needs (SEN) and compares the degree of behavioral change between students with and without SEN. Additionally, the study examines students’ perceptions of the GBG. An ABAB reversal design with a follow-up phase was employed to evaluate the intervention’s effects. The study included six participants: three students with SEN who exhibited disruptive behaviors, aged 11 to 14, and three students without SEN who also exhibited disruptive behaviors, aged 11 to 13. The results indicate that the GBG is highly effective in reducing disruptive behaviors, with similar levels of improvement observed in both groups. The social validity findings highlight that students found the GBG enjoyable and engaging. The study discusses its limitations and provides practical implications for educators.

## 1. Introduction

Addressing students’ academic and social unreadiness for school is among the most significant challenges faced by teachers (Schwartz, 2019). This lack of readiness disrupts teaching and learning processes, often leading to disruptive behaviors (Flower et al., 2014a). These behaviors can range from severe or dangerous, such as aggression toward peers or self-injurious behavior, to less hazardous forms (T. E. Smith et al., 2022). Regardless of the type and severity, disruptive behaviors, especially when frequent and prolonged, adversely affect educational settings (Vargo et al., 2014).

Disruptive behaviors often have negative impacts on teachers’ ability to teach and students’ learning skills (Okeke et al., 2023). Teachers frequently report that such behaviors undermine the quality of the classroom environment and their professional satisfaction (Office for Standards in Education, Children’s Services, and Skills, 2014, 2019). With legislation mandating students with special educational needs (SEN) be placed in the least restrictive educational settings, students displaying disruptive behaviors are more likely to be placed in general education classrooms (Ministry of National Education, 2023). Consequently, all teachers must be equipped to provide behavioral support alongside academic instruction (Flower et al., 2014a). As teachers’ lack of behavior management skills can exacerbate these challenges, it is crucial to adopt efficient and practical classroom management strategies that require minimal time and resources to learn and implement (Groves et al., 2022). One such strategy is the Good Behavior Game.

The Good Behavior Game (GBG) is a classroom management strategy used for over half a century, with substantial empirical research supporting its effectiveness (Jornevald et al., 2023). The strategy consists of several key components. First, students are divided into teams to foster cooperation and encourage adherence to classroom rules. Clear behavioral expectations are established to ensure students understand the specific, observable, and measurable behaviors required, such as remaining seated and raising hands before speaking. A monitoring and scoring system allows teachers to track rule violations or positive behaviors, awarding points accordingly. Reinforcement plays a crucial role, as teams that meet behavioral goals receive incentives such as privileges, praise, or small rewards. Immediate and consistent feedback helps students to associate their behaviors with outcomes, reinforcing expectations effectively. Over time, external reinforcements gradually fade as students internalize appropriate behaviors, promoting long-term improvements (Flower et al., 2014b). Research consistently demonstrates that the GBG is effective in reducing disruptive behaviors and enhancing classroom engagement, making it a valuable tool for educators seeking an evidence-based behavioral strategy (Kellam et al., 2011).

The effectiveness of the GBG can be understood through the lens of operant conditioning (Skinner, 1953), which explains how behaviors are learned and maintained through reinforcement. The GBG utilizes positive reinforcement by rewarding teams for demonstrating appropriate behaviors, increasing the likelihood that students will repeat these behaviors. By consistently reinforcing rule-following behaviors and providing immediate feedback, the GBG strengthens students’ ability to self-regulate over time. Furthermore, as the intervention progresses, extrinsic rewards gradually decrease, promoting the internalization of positive behaviors. This process aligns with Skinner’s principles, demonstrating how structured reinforcement contingencies shape and maintain student behavior in classroom settings.

The first research on the GBG was conducted by Barrish et al. (1969), who examined its effectiveness in reducing disruptive behaviors among fourth-grade students during academic activities. The findings indicated that the GBG was effective in reducing disruptive behaviors. Since then, numerous studies have demonstrated the effectiveness of the GBG across various grade levels, from kindergarten (Donaldson et al., 2015) to high school (Flower et al., 2014b), and in diverse school settings, including general education (Bohan et al., 2022) and special education (Groves et al., 2022).

Systematic reviews and meta-analyses have yielded mixed results regarding the effectiveness of the GBG. Flower et al. (2014a) conducted a systematic review revealing that the GBG effectively reduced disruptive behaviors. Similarly, Bowman-Perrott et al. (2016) reported in their meta-analysis that the GBG strategy reduced students’ disruptive behaviors while increasing positive social behaviors. However, S. Smith et al. (2019) conducted a meta-analysis indicating that the GBG strategy had moderate effectiveness on target behaviors compared to the prior studies and it was found ineffective in addressing students’ timid behaviors. Jornevald et al. (2023) conducted a systematic review reporting that the GBG positively impacted the target behaviors of many students with SEN in inclusive education settings. However, the precision of results for students with severe disruptive behaviors was lacking. Additionally, few studies have examined the effectiveness of the GBG in reducing disruptive behaviors among students with neurodevelopmental disorders such as attention deficit hyperactivity disorder and autism spectrum disorder (ASD).

The GBG has been widely studied as a classroom management strategy to reduce disruptive behaviors and promote positive social interactions. Systematic reviews and meta-analyses have provided mixed results regarding its effectiveness. While some studies indicate significant reductions in disruptive behaviors (Flower et al., 2014a; Bowman-Perrott et al., 2016), others suggest only moderate effects, particularly in the treatment of severe disruptive behaviors (S. Smith et al., 2019; Jornevald et al., 2023). A critical gap in the literature is the limited research on the impact of the GBG on students with neurodevelopmental disabilities, such as ASD, particularly in inclusive educational settings. Given the increasing emphasis on inclusive education, it is important to examine how the GBG affects students with and without SEN who exhibit disruptive behaviors. The need for such a study arises from these groups’ different behavioral and learning characteristics. To fill this gap, a study is needed that systematically examines and compares the effects of the GBG on disruptive behaviors in students with and without SEN. This comparison is particularly important because students with SEN may respond differently to the GBG due to their unique cognitive, social, and emotional characteristics. Determining whether the GBG is equally, more, or less effective for these groups will provide educators and policymakers with important insights into classroom behavior management strategies. Understanding these differential effects will help to develop interventions to better support learners with different backgrounds in inclusive settings. By addressing this research gap, the study contributes to the growing literature on evidence-based classroom management practices and will inform future adaptations of the GBG for students with diverse needs. The following questions were addressed in this study: (a) What is the effect of the GBG on the level of disruptive behavior among students with SEN? (b) Does the GBG reduce disruptive behaviors of students with SEN to a level comparable to that of their typically developing peers with disruptive behaviors? (c) What are the students’ opinions regarding implementing the GBG strategy?

## 2. Method

The study was approved by an Institutional Review Board, and necessary permissions were obtained from the school district to ensure adherence to ethical standards. Parents of participating students were informed about the study, and their written consent was obtained. To maintain participant anonymity, pseudonyms were used throughout the study.

### 2.1. Participants

#### 2.1.1. Students with Special Education Needs

Three students aged between 11 and 14 who received inclusive education were included in the study. The students were identified based on teacher observations. All students exhibited a high rate of disruptive behavior during independent work. Ali was a 12-year-old 5th grade student diagnosed with autism spectrum disorder. He often called out to his teacher and talked to his friends without permission. Ege was an 11-year-old 6th grade student diagnosed with specific learning disabilities. He frequently engaged in disruptive behaviors and needed constant reminders from his teacher to stay on task and complete the activity. Kadir was a 14-year-old 8th grade student diagnosed with an intellectual disability. He often did not sit properly in his seat during class and talked to his friends without permission. The students were Turkish.

#### 2.1.2. Students Without Special Education Needs

Two male and one female Turkish students with disruptive behaviors attended the same classes as the students with SEN. The ages of these students ranged between 11 and 13 years. None of the students had an identified disability. To identify these students, the teachers were asked to name the three students who exhibited the highest frequency of disruptive behavior in their classes and the disruptive behavior they frequently exhibited. Based on the teachers’ responses, the students were invited to participate in the study. Ahmet was an 11-year-old 5th grade student who often talked to his friends without permission from the teacher. Esma, a 12-year-old 6th grade girl, and Kutay, a 13-year-old 8th grade boy, frequently exhibited behaviors such as not sitting in their seats, talking to their friends without permission, swaying at their desks, and waiting with their heads on their desks.

### 2.2. Interventionist

The second author of the study carried out the intervention process. She was the students’ science teacher and held bachelor’s and master’s degrees in science education and a master’s degree in special education. Currently pursuing a Ph.D. in special education, the interventionist has eight years of teaching experience in inclusive classrooms.

### 2.3. Setting

The study was conducted in three classrooms of an inclusive elementary school located in a northern province of Türkiye during the 2021–2022 academic year. The school served 275 students, including those with SEN. The average class size was 23 students (range = 15–34). The majority of the students at the school were Turkish and came from low to middle socio-economic backgrounds. The school did not implement a specific classroom management system in any of its classes. Data were collected through observation conducted during science classes, where students were engaged in teacher-supported tasks. Science classes were held four hours per week across all grade levels, typically scheduled as two sessions per week, each lasting two hours. Observations were collected twice a week, with one session conducted per day.

### 2.4. Response Definitions and Measurement

Target behaviors were identified based on input from working with the target students in their classrooms and the interventionist. *Verbal disruption* included talking to the teacher or friends without permission, making noise by tapping the desk/table with their hands, feet, or a pen, or shouting. *Inappropriate seating behavior* was defined as actions such as shaking the chair or desk, the student’s feet or desk legs not touching the floor for at least 3 s, the student’s buttocks not touching the flat part of the chair for more than 1 s, or the student being out of their seat and leaning over the table. *Off-task behavior* was defined as the student failing to face the teacher, activity materials, or the board for at least 3 s, or not engaging with writing activities by having their pencil away from the notebook or activity paper for at least 3 s during writing activities. Data were collected separately for each behavior topography defined above but were combined into a single measure, referred to as “disruptive behavior,” during the analysis. In other words, the disruptive behavior was coded when any of the above behaviors were exhibited at the end of the observation interval.

Observations were conducted during the last 20 min of science class, a period designated for independent work. A momentary time sampling procedure was utilized for data collection. Observation sessions were divided into 1 min intervals. At the end of each interval, a sound played through the observer’s headphones, prompting her to observe the target students for 3 s. At the end of each observation interval, the observer recorded whether the target students exhibited disruptive behaviors. Data were calculated as the percentage of intervals in which a disruptive behavior occurred.

### 2.5. Experimental Design

The current study employed an ABAB reversal design with a follow-up phase, a single-case experimental research (SCER) design, to evaluate the effects of the GBG on the disruptive behaviors of students with and without SEN. SCER designs are particularly useful for examining functional relationships between an intervention and target behaviors (Ledford & Gast, 2018). In this study, the ABAB design involved the repeated implementation (B phase) and withdrawal (A phase) of the independent variable (the GBG) to establish experimental control. Phase changes occurred when stable data on disruptive behavior were observed for students with SEN. Additionally, follow-up observations were conducted 2 and 4 weeks after the completion of the second B phase to assess the maintenance of the intervention effect on students’ disruptive behaviors.

### 2.6. Procedures

#### 2.6.1. Baseline

Baseline sessions were conducted in all three classrooms with the target students. The sessions consisted of the typical routine of a science class. During these sessions, the typical science class routine was followed, and no additional classroom management systems or strategies were implemented. When students exhibited disruptive behaviors, the interventionist provided standard reminders (e.g., “You have to raise your hand to speak”) and continued the lesson as usual.

#### 2.6.2. Good Behavior Game

The interventionist introduced the GBG by explaining to the class that they would play a team-based game with a prize awarded at the end of the lesson. She clarified that the teams would need to follow specific rules to win the game. Before starting, the interventionist outlined four rules: (a) raise your hand to speak, (b) sit at your desk, (c) work quietly, and (d) keep your hands, feet, and materials to yourself. To ensure understanding, the interventionist provided examples and non-examples of each rule and asked students to offer their own examples. The rules were written on the board, and the interventionist explained that she would monitor students during the task. If a target student failed to follow a rule, their team would receive “−” point, while teams adhering the rules would receive a “+” point. The interventionist also explained that the total number of “−” points received would be subtracted from the total number of “+” points to determine each team’s final score. The teams earned “+” or “−” points based on the target students’ engagement in disruptive behaviors. Team(s) that reached or exceeded a specific threshold of “+” points would win a surprise at the end of the game. To maintain student attention and motivation, the interventionist adjusted the winning criterion based on group performance and announced it at the end of each session.

The interventionist divided the class into two teams with an average of 13 students per team (range = 11–17). Team members remained consistent throughout the intervention and target students with and without SEN were placed in separate teams. The interventionist asked each team to choose a superhero name (e.g., Batman or Superman) and wrote the team names on the board. Team members sat together in designated areas. The interventionist used a timer on her phone to signal every 1 min interval. At the end of each interval, she observed the target students for 3 s. Observations alternated between target students, following a consistent row-by-row pattern. The games were played during the final 20 min of the independent activity portion of the lesson. Each game ended at least 5 min before the lesson concluded to allow time for awarding the prize. At the end of each game, the interventionist announced the winning team(s) and distributed the prizes. Both teams were rewarded if they met or exceeded the winning criteria. Prizes were selected by the teacher and included rewards such as extra break time, snacks, or extra quiz points.

#### 2.6.3. Reversal Phase

The GBG was withdrawn when there was a favorable change in the disruptive behaviors of the target students with SEN compared to baseline levels. The main purpose of the reversal phase was to demonstrate that the GBG was the cause of the change in student behavior. By withdrawing the intervention and observing whether disruptive behaviors return to baseline levels, researchers can establish a functional relationship between the intervention and the observed behaviors. During this phase, the interventionist resumed typical classroom practices as conducted during the baseline phase, without implementing the GBG strategy.

#### 2.6.4. Follow-Up Phase

To assess the maintenance of behavioral changes, follow-up observations were conducted intermittently at 2 and 4 weeks after the conclusion of the second intervention phase (B phase). These observations aimed to determine whether the target students maintained improvements in their target behaviors over time without ongoing implementation of the GBG. The follow-up phase was conducted under baseline/reversal conditions, with the interventionist engaging in regular classroom activities but without reintroducing any new variables or procedures.

#### 2.6.5. Inter-Observer Agreement

The first author collected inter-observer agreement (IOA) data for at least 20% of the sessions in each phase. The IOA was calculated based on the observation intervals by determining the intervals where the observer and the interventionist agreed or disagreed. The reliability coefficient was then calculated by dividing the number of intervals in which the observers agreed by the total number of intervals in an observation and multiplying the result by 100. The mean IOA coefficient for verbal disruption was 97% (range = 89–100%). For inappropriate seating behavior, the mean IOA coefficient was 91% (range = 83–97%). The mean IOA coefficient for off-task behavior was 94% (range = 91–97%).

#### 2.6.6. Treatment Integrity

The first author also collected treatment integrity data for at least 20% of the sessions in each phase. The steps included in the treatment integrity form were as follows: (a) announcing the start of the game, (b) writing the team names on the board, (c) directing team members to sit together, (d) writing the classroom rules on the board and reviewing them with the students, (e) reminding students about the achieving criteria (i.e., the team with the highest score wins; this marks the start of the game, timer, and point allocation), (f) identifying and recording the target students who followed and did not follow the rules (data were collected only for the target student in each group) (g) recording the behaviors of the target students, (h) subtracting the “−” numbers from the “+” numbers of the teams to determine the remaining “+” number, (i) announcing the criteria of the day and the winning team(s), and (j) awarding the winning team(s). Each step was coded as “completed” or “not completed.” The treatment integrity coefficient was calculated by dividing the number of steps correctly performed by the interventionist by the total number of steps and multiplying the results by 100. The treatment integrity coefficient was 100% across all participants and phases.

### 2.7. Data Analysis

#### 2.7.1. Visual Analysis

Data on participants’ disruptive behaviors were visualized using line graphs after each observation session. The effects of the GBG on disruptive behaviors were evaluated by conducting visual analyses of the graphed data. Visual analysis involves examination of the level, trend, variability, overlap, immediacy of effect, and consistency of data patterns within and across baseline and intervention phases (Kratochwill et al., 2010). Visual analysis was also used to inform decisions regarding phase changes. To quantify behavioral change, the averages of data points within each phase were calculated and compared. Behavioral changes were categorized as follows: low level for mean differences less than 30%, medium level for differences between 30% and 60%, and high level for differences exceeding 60%.

#### 2.7.2. Effect Size

The Tau-*U* method was used to calculate the effect size. This method controls for a therapeutic trend in the baseline phase. A web-based calculator was used to compute Tau-*U* values (http://www.singlecaseresearch.org, accessed on 3 February 2024). Tau-*U* scores range between 0–1 and are interpreted as follows: scores between 0 and 0.65 suggest weak effects, scores between 0.66 and 0.92 suggest medium effects, and scores between 0.93 and 1.0 indicate large or strong effects (Rakap, 2015).

### 2.8. Social Validity

Social validity data were collected at the conclusion of the study from the target students with and without SEN. The social validity data were collected using the social validity questionnaire developed by Groves et al. (2022). The questionnaire included six questions with response options of “yes”, “no”, and “maybe”, as well as two open-ended questions to gather qualitative feedback. The data were analyzed descriptively.

## 3. Results

### 3.1. Effectiveness

**Figure 1:**
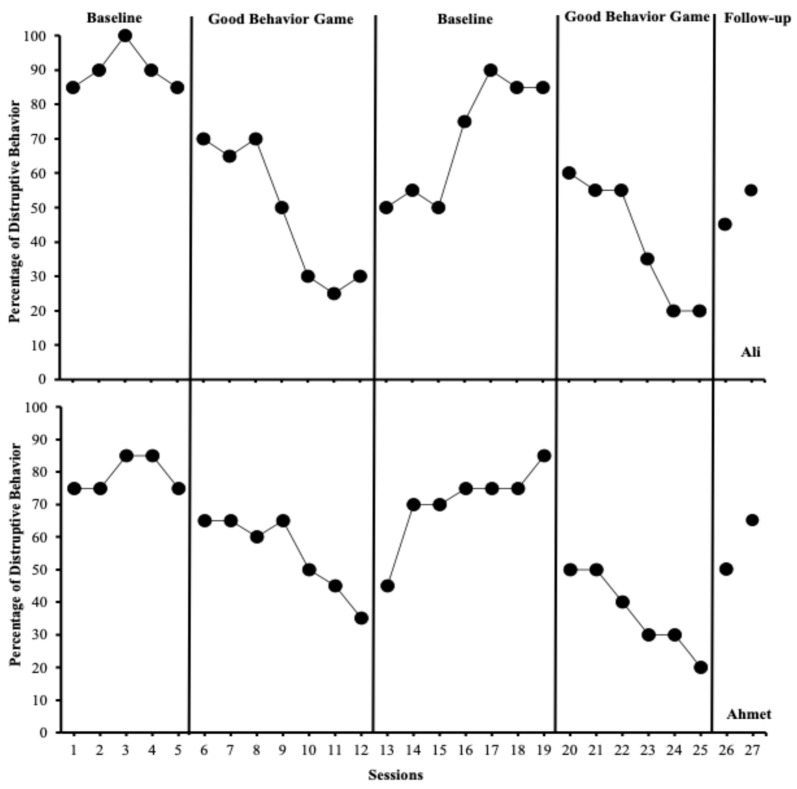
Percentage of intervals with disruptive behaviors across Ali (students with special educational needs; top panel) and Ahmet (typically developing peer; bottom panel).

Figure 1 illustrates the percentage of intervals with disruptive behaviors for Ali (students with SEN; top panel) and Ahmet (students without SEN; bottom panel). During the initial baseline phase, Ali (M = 90%; range = 85–100%) and Ahmet (M = 79%; range = 75–85%) exhibited high levels of disruptive behavior. Following the introduction of the GBG in session 6, Ali’s disruptive behaviors decreased significantly (M = 48%; range = 25–70%), as did Ahmet’s (M = 55%; range = 35–65%). Both students demonstrated moderate levels of disruptive behavior during the initial GBG sessions. When the GBG was withdrawn in session 13, both Ali (M = 70%; range: 50–90%) and Ahmet’s (M = 71%; range: 45–85%) disruptive behaviors increased substantially, returning to high levels. Upon reintroducing the GBG in session 20, Ali (M = 40%; range: 20–60%) and Ahmet’s (M = 36%; range: 20–50%) disruptive behaviors significantly decreased, showing moderate levels of disruptive behavior. During the maintenance phase, Ali exhibited disruptive behaviors at 45% and 55% in weeks 2 and 4, respectively, while Ahmet’s disruptive behaviors were recorded at 50% and 65%. Although both students demonstrated moderate levels of disruptive behavior in the maintenance phase, Ali maintained his behavioral change better than Ahmet.

**Figure 2:**
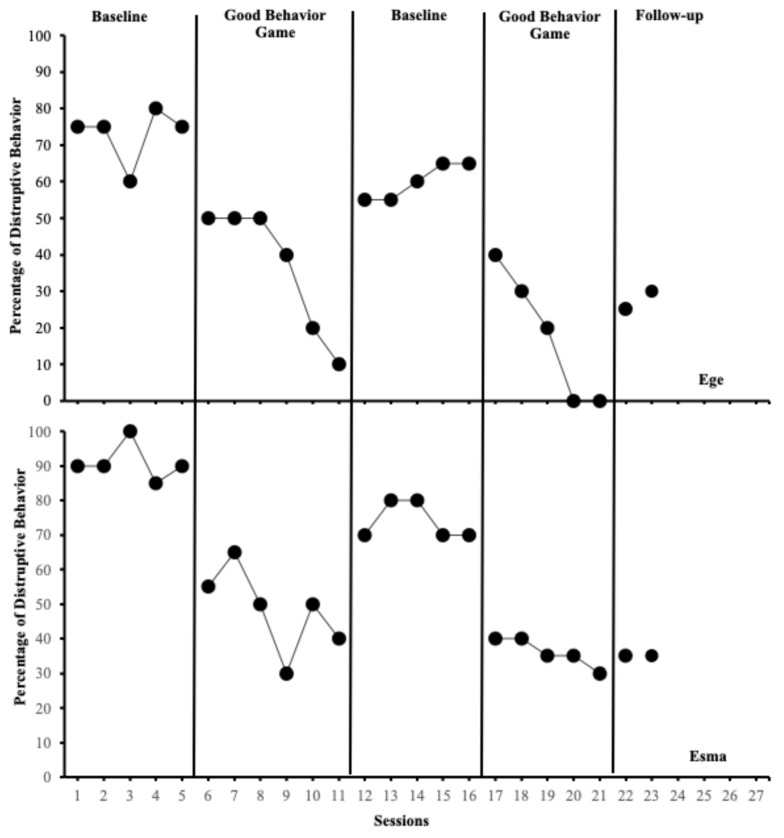
Percentage of intervals with disruptive behaviors across Ege (students with special educational needs; top panel) and Esma (typically developing peer; bottom panel).

Figure 2 presents the percentage of intervals with disruptive behaviors for Ege (students with SEN; top panel) and Esma (students without SEN; bottom panel). During the baseline phase, Ege (M = 73%; range = 60–80%) and Esma (M = 91%; range = 85–100%) displayed high levels of disruptive behavior. Following the implementation of the GBG in session 6, Ege (M = 37%; range = 10–50%) and Esma’s (M = 48%; range = 40–65%) disruptive behaviors reduced significantly. Both students exhibited moderate levels of disruptive behavior during the first GBG sessions. When the GBG was withdrawn at session 12, Ege (M = 60%; range: 55–65%) and Esma’s (M = 74%; range: 70–80%) disruptive behaviors increased, returning to high levels. After reintroducing the GBG in session 17, Ege’s disruptive behaviors decreased to low levels (M = 18%; range = 0–40%), whereas Esma’s disruptive behaviors decreased to moderate levels (M = 36%; range = 30–40%). During the maintenance phase, Ege’s disruptive behaviors were recorded at 25% and 30% in weeks 2 and 4, while Esma maintained consistent behavioral change at 35%. Both students demonstrated sustained improvements in their behavior.

**Figure 3:**
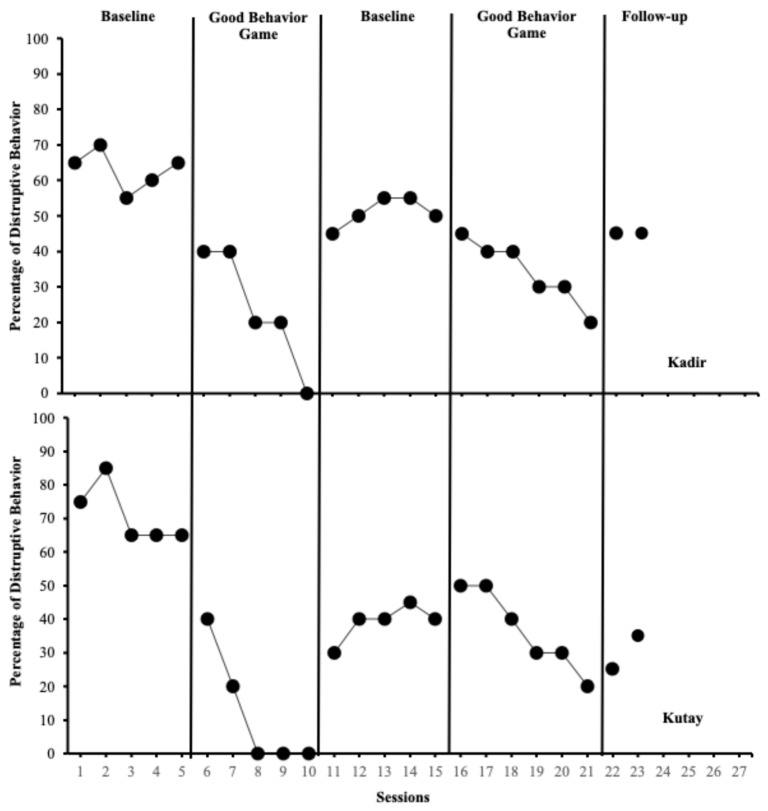
Percentage of intervals with disruptive behaviors across Kadir (students with special educational needs; top panel) and Kutay (typically developing peer; bottom panel).

Figure 3 depicts the percentage of intervals with disruptive behaviors for Kadir (students with SEN; top panel) and Kutay (students without SEN; bottom panel). In the baseline phase, Kadir (M = 63%; range = 55–70%) and Kutay (M = 71%; range = 65–85%) exhibited high levels of disruptive behavior. With the introduction of the GBG in session 6, both Kadir (M = 24%; range = 0–40%) and Kutay’s (M = 12%; range = 0–40%) disruptive behaviors reduced to low levels. Following the withdrawal of the GBG in session 11, Kadir (M = 51%; range = 45–55%) and Kutay’s (M = 39%; range = 30–45%) disruptive behaviors increased to moderate levels. After reintroducing the GBG in session 16, both students displayed moderate levels of disruptive behavior, with Kadir (M = 34%; range = 20–45%) and Kutay’s (M = 37%; range = 20–50%) disruptive behaviors significantly reduced compared to the withdrawal phase. During the maintenance phase, Kadir exhibited disruptive behaviors at 45% in weeks 2 and 4, while Kutay demonstrated consistent improvement, recorded at 35% in both weeks. Both students maintained their behavioral changes effectively.

The comparisons between baseline and intervention (AB_1_) and withdrawal and reimplementation (AB_2_) suggest that the GBG was very effective at reducing disruptive behaviors for Ege (Tau-*U_AB_*_1_ = 1; Tau-*U_AB_*_2_ = 1, respectively), Esma (Tau-*U_AB_*_1_ = 1; Tau-*U_AB_*_2_ = 1, respectively), and Kadir (Tau-*U_AB_*_1_ = 1; Tau-*U_AB_*_2_ = 0.97, respectively). For Ali, the comparison between baseline and intervention suggests that the GBG was very effective at reducing disruptive behaviors (Tau-*U_AB_*_1_ = 1); however, the comparison between withdrawal and reimplementation indicates a weak effect (Tau-*U_AB_*_2_ = 0.62). Similarly, for Ahmet, the comparison between baseline and intervention indicates that the GBG was highly effective at reducing disruptive behaviors (Tau-*U_AB_*_1_ = 1), while comparison between withdrawal and reimplementation for disruptive behaviors suggest a medium effect (Tau-*U _AB_*_2_ = 0.90). For Kutay, the comparison between baseline and intervention suggests that the GBG was very effective at reducing disruptive behaviors (Tau-*U_AB_*_1_ = −1). However, the comparison between withdrawal to reimplementation indicates no observed effect (Tau-*U_AB_*_2_ = 0.10).

### 3.2. Social Validity Results

Table 1 displays participants’ responses to the social validity questionnaires. The students reported that they enjoyed playing the GBG, found the rules easy to understand, and felt it was fair that everyone had to follow them. They also mentioned that the GBG made it easier for them to complete their schoolwork. All students, except Kutay, indicated that the GBG helped them to regulate their behavior and expressed a desire to continue playing the game. While all students stated that the best part of the GBG was winning prizes, four students mentioned that their least favorite part was not winning prizes. Two students did not respond to the question about the least favorite part of the GBG.

## 4. Discussion

The purpose of this study was to investigate the effectiveness of the GBG in reducing disruptive behaviors among students with SEN and to compare the degree of behavioral change between students with and without SEN. Additionally, the study sought to explore students’ perceptions of the GBG. The findings suggest that the GBG effectively reduces disruptive behaviors in students with SEN, with behavioral changes comparable to those observed in their peers. The social validity results further indicate that students enjoy playing the GBG, with winning prizes being a particularly motivating factor. These findings align with previous research on the effectiveness of the GBG for different student groups, including those with special needs and those exhibiting disruptive behaviors (e.g., Flower et al., 2014b; Groves & Austin, 2020; Groves et al., 2022; Moore et al., 2022).

The results revealed that the most pronounced behavioral changes occurred during the initial baseline and implementation phases of the GBG, while changes during the reversal and subsequent re-implementation phases were less marked. This is consistent with the findings of the studies by Sewell (2020) and Moore et al. (2022). Several factors may explain these results. First, structured interventions like the GBG may initially captivate students due to their novelty, leading to heightened motivation and compliance with the rules. Second, while students responded positively to the initial introduction of the GBG, they may have exhibited less enthusiasm during the re-implementation phase. Third, the effectiveness of rewards may have diminished over time as their reinforcing value decreased. Finally, reduced frequency of gameplay may have contributed to a decline in student motivation, leading to a moderate impact of the GBG during subsequent phases.

During the baseline and reversal phases, disruptive behaviors accounted for over 53% (range = 34–90%) of the observation intervals. Such high levels of disruptive behavior are known to significantly reduce instructional time (Sutherland et al., 2002) and hinder learning. Furthermore, disruptive behaviors often prevent students from thriving in inclusive school settings (Strain et al., 2011) and require substantial time and effort from teachers to manage (Hume et al., 2014). The findings indicate that the GBG successfully reduces disruptive behaviors, creating conditions that improve students’ access to academic instruction.

Interestingly, the results suggest that the GBG has a similar effect on reducing disruptive behaviors in students with and without SEN who exhibit intense disruptive behaviors. This finding extends the existing literature, as no previous studies have directly compared these groups. Several factors may explain this result. First, the structured nature of the GBG, with its clear rules and expectations (Jornevald et al., 2023), likely contributed to its consistent effectiveness across both groups. Second, the game may have fostered teamwork, peer support, and a sense of belonging in the classroom, which positively influenced all participants. Third, the GBG’s emphasis on monitoring and reinforcing positive behaviors likely provided a robust framework for behavioral change, especially for students with disruptive tendencies. Consistent reinforcement helps to sustain desired behaviors and promotes long-term behavioral improvements. Finally, by focusing on prosocial behaviors rather than merely punishing negative actions, the GBG likely cultivated a positive classroom culture, offering students with behavioral difficulties examples of appropriate behaviors to emulate (Coombes et al., 2016).

The social validity findings highlight the GBG as a sustainable intervention for managing disruptive behaviors. Student responses to the social validity assessments indicated overwhelmingly positive perceptions of the GBG. These findings are consistent with prior studies reporting positive social validity outcomes (e.g., Moore et al., 2022; Groves & Austin, 2020). Anecdotal evidence further supports these results. During the reversal phase, students frequently inquired about the absence of the GBG and expressed a desire to resume playing. Teachers also noted that students requested to play the GBG in their classes. When students were informed that prizes were unavailable due to their non-participation in the GBG, they eagerly asked when the game would be reintroduced. Such comments demonstrate not only the students’ acceptance of the GBG procedures but also their enthusiasm for its continuation.

### 4.1. Limitations and Recommendations for Future Research

When interpreting the results of the current study, readers should consider several limitations. First, the small sample size limits the generalizability of the findings. The study included a narrow group of participants, making it difficult to conclude whether similar effects would occur in students with other special needs or in different educational settings. Future research with larger and more diverse samples is needed to address this limitation and to explore the potential for broader applicability of the GBG. Second, the study focused exclusively on disruptive behaviors and did not assess on-task behaviors. While the reduction of disruptive behaviors is a critical outcome, understanding the impact of the GBG on students’ task-related behaviors would provide a more comprehensive evaluation of its effectiveness. Future studies should collect data on both disruptive and task-related behaviors to better capture the full range of behavioral change associated with the intervention.

Third, the use of 1 min intervals in the time sampling procedures may have introduced limitations in the accuracy and sensitivity of the data. Shorter intervals (e.g., 20 or 30 s) are more likely to detect subtle changes in student behavior and provide a more precise representation of behavioral trends. For example, continuous recording or shorter interval sampling (e.g., 10 s intervals) could better capture fluctuations in behavior and improve the reliability of the observations. Specifically, the use of 1 min intervals for partial interval recording of disruptive behaviors may have reduced the sensitivity of the data and limited the study’s ability to detect smaller, yet meaningful, changes in behavior. Additionally, the study was conducted in a single inclusive elementary school within a specific cultural and educational context, which represents a limitation in terms of generalizability. While this extends GBG research to a setting outside of the United States, an important contribution, it is important to note that the findings may be specific to this school and its unique characteristics. The majority of the students at the school were Turkish and came from low to middle socio-economic backgrounds. Moreover, the school did not implement a specific classroom management system in any of its classes. Additional research is needed to replicate the findings in other schools and cultural environments to assess the generalizability of the GBG’s effectiveness. Lastly, while the social validity data suggest that students found the GBG enjoyable and engaging, the study relied primarily on self-reported measures and anecdotal evidence. Future studies could incorporate more robust measures of social validity, such as teacher and parent feedback, to provide a more comprehensive understanding of the intervention’s acceptability and sustainability.

### 4.2. Implications for Practice

Despite its limitations, the results of this study emphasize several important implications for educational practice. The GBG has been described as a “behavioral vaccine” that supports learning for all students (Embry, 2002). The findings of this study indicate that the GBG is an effective strategy for reducing disruptive behaviors in both students with special educational needs (SEN) and their typically developing peers. By fostering a structured and positive classroom environment, the GBG promotes behavioral improvement and enhances students’ access to instructional time.

The GBG encourages teachers to clearly define expectations and provide consistent, high levels of reinforcement for appropriate behaviors. This approach not only supports the behavioral and academic needs of students with SEN but also benefits all students in the classroom by creating a more cohesive and supportive learning environment. Teachers can integrate the GBG into their existing classroom management strategies to enhance student engagement, reduce disruptions, and promote positive social interactions among peers.

Furthermore, the GBG’s flexibility allows it to be adapted across various educational contexts, making it a versatile tool for inclusive education settings. Its emphasis on teamwork and peer support can foster a sense of belonging and cooperation among students, which is especially valuable in classrooms with diverse needs. Teachers are encouraged to implement the GBG consistently and to involve all students in the process, ensuring that every student has an opportunity to participate and benefit from the intervention.

To maximize the effectiveness of the GBG, teachers should consider using clear, achievable goals and providing immediate feedback and rewards to reinforce positive behaviors. Additionally, incorporating student input when designing classroom rules and reward systems may increase student motivation and commitment to the game. By focusing on consistent application of the GBG, educators can create a proactive approach to behavior management that promotes a positive and productive classroom culture.

## Figures and Tables

**Table 1:** Results of participants’ social validity questionnaires.

Statement	Number of Responses		
	Yes	Maybe	No
Did you enjoy playing the good behavior game?	3(2)	0(1)	0(0)
Is your behavior better when you play the GBG?	3(1)	0(1)	0(1)
Does playing the game help you to get more schoolwork done?	2(2)	1(1)	0(0)
Did you find the rules of the game easy to understand?	2(3)	1(0)	0(0)
Everyone in your team has to follow the rules of the game to earn points. Do you think this is fair?	3(3)	0(0)	0(0)
Would you like to continue playing the game in class?	3(2)	0(0)	0(1)
What did you like best about playing the game?	Receiving a reward 3(3)		
What is your least favorite part of playing the game?	Not earning reward 1(3) No answer 2(0)		

Note: Numbers in parentheses indicate students without special educational needs responses.

## Data Availability

Electronic data summaries of raw data are available upon request.
